# Neurometabolic Diagnosis in Children who referred as Neurodevelopmental Delay (A Practical Criteria, in Iranian Pediatric Patients)

**Published:** 2016

**Authors:** Parvaneh KARIMZADEH, Narjes JAFARI, Habibeh NEJAD BIGLARI, Sayena JABBEHDARI, Simin KHAYAT ZADEH, Farzad AHMAD ABADI, Azra LOTFI

**Affiliations:** 1 Pediatric Neurology Research Center, ShahidBeheshti University of Medical Sciences, Tehran, Iran; 2 Pediatric Neurology Department, Mofid Children’s Hospital, Faculty of Medicine, ShahidBeheshti University of Medical Sciences, Tehran, Iran; 3 Pediatric Neurologist, Ardabil University of Medical Sciences, Ardabil, Iran

**Keywords:** Clinical findings, Neurometabolic disorders, Children, Developmental delay, Seizure

## Abstract

**Objective:**

We aimed to investigate the clinical and para clinical manifestations of neuro metabolic disorders, in patients who presented by neuro developmental delay in their neuro developmental milestones.

**Materials & Methods:**

The patients diagnosed as neuro developmental delay and regression with or without seizure at the Neurology Department of Mofid Children Hospital in Tehran, Iran between 2004 and 2014 were included in our study. These patients diagnosed as neuro developmental delay by pediatric neurologists in view of diagnostic /screening neuro developmental assessment tests. The patients who completed our inclusion criteria as neuro metabolic disorders were evaluated in terms of metabolic and genetic study in referral lab.

**Results:**

Overall, 213 patients with neurometabolic disorders were diagnosed. 54.3% of patients were male. The average age of patients was 41 +-46.1 months. 71.4% of parent’s patients had consanguinity of marriages. Eighty seven percent of patients had developmental delay (or/and) regression. 55.5% of them had different type of seizures. Overall, 213 patients with 34 different neurometabolic disorders were diagnosed and classified in the 7 sub classes, consisting of: 1- organic acidemia and aminoacidopathy (122 patients), 2-storage disease (37 patients) 3- eukodystrophy (27 patients), other classes consisted: lipid oxidation disorders, urea cycle disorders, progressive myoclonic epilepsy; and peroxizomal disorders (27 patients).

**Conclusion:**

In patients with developmental delay or regression, with or without seizure, abnormal neurologic exam along with positive family history of similar disorder or relative parents, abnormal brain imaging with specific patterns, neurometabolic disorders should be considered as one of the important treatable diseases

## Introduction

The incidence of autosomal recessive neurometabolic disorders is relatively high in Iran, probably due to the frequency of consanguineous marriages. Reevaluation of patients with delayed neurodevelopmental milestones in a referral neurometaboliccenter is very important. Early detection and early intervention of patients with neuro developmental delay/ regression as treatable neuro metabolic disorder can cause prevention of more brain insults during early infants and childhood. There are few beneficial clinical criteria for differentiating neuro metabolic disorders (2, 3). Most of the time, the presenting symptoms are nonspecific (4-6). Morbidity and mortality is the main result of neurodegenerative and neuro metabolic disorders in children. In this study we report complete practical criteria on children with developmental delay/regression with or without seizure as neuro metabolic cases referred to the Pediatric Neurology Research Center of Mofid Children’s Hospital, Tehran, Iran.

## Materials & Methods

This diagnostic study was performed on patients referred to the Neurology Department of Mofid Children’s Hospital in Tehran, Iran with neuro developmental delay and regression with or without seizure or other items that predisposed them to metabolic disease. The diagnosis was done based on clinical manifestation, laboratory assessment and neuro imaging findings. The demographic data of patients were gathered as age, gender, developmental status, past medical history, and clinical and neuro imaging findings. We applied practical criteria for our patients in view of elementary diagnosis and then evaluated them by metabolic /genetic study for confirming of our diagnosis. The neuro metabolic disorders diagnosis was confirmed based on the type of disorder including acylcarnitine profile assessment in lipid oxidation disorders; enzyme assessment in Sandhoff and Tay–Sachs disease; biotinidase deficiency (BD); metachromatic leukodystrophy (MLD) and galactosemia; very long chain fatty acid (VLCFA) assessment in peroxisomal disorders; serum acyl carnitine profile assessment and urine organic acid in organic acidemia; and genetic assessment in plezeousmerzbacher disease (PMD), PMD-like and for confirmation of other neurometabolic disorders. In the radiological assessment, location and pattern of white matter involvement (pre-ventricular involvement with anterior or posterior dominancy, with or without u-fiber involvement, leukodystrophic patternor myelination delay), brain edema, brain atrophy (generalized; cortical atrophy; cerebellum atrophy; or sylvan groove opening due to fronto temporal atrophy), hemorrhage (parenchymal hemorrhage, subdural hematoma, or stroke), basal ganglia involvement (globuspallidus, putamen, caudate nucleus), thalamus, cyst, subdural effusion, axial hydrocephaly, heterotopia, cortical malformation, MRS involvement, and corpus callosum agenesia were assessed. Institutional ethical approval was obtained from the Pediatric Neurology Research Center of ShahidBeheshti University of Medical Sciences, Tehran, Iran. All parents signed a written consent for participation in this study. The data were analyzed using SPSS (Chicago, IL, USA).

## Results

**Fig1:**
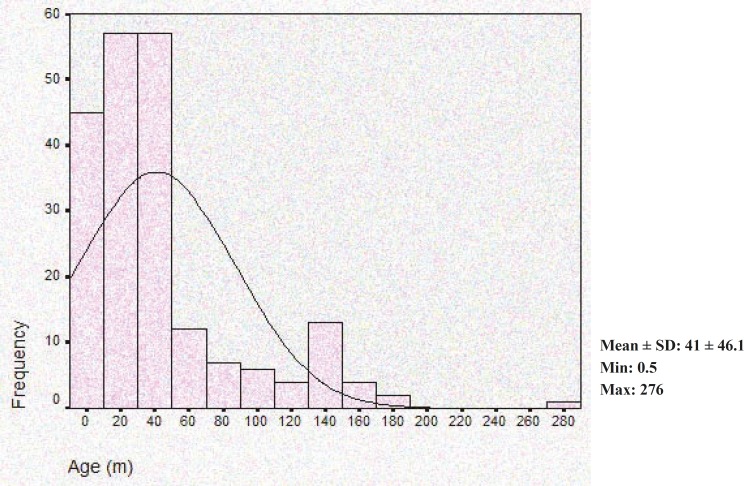
Distribution of patient’s age in our study

Overall, 213 patients with 34 different neurometabolic disorders were diagnosed. A total of 54.3% were male. The median age of patients at detection time was 41 +- 46.1 months (Figure 1). A total of 23.5% of patients had a positive family history of similar diseases. 71.4% of patients were offspring of consanguineous marriages as follows: 57.4% were first degree and 14% were second degree. Patients were classified in 7 classes as follows: 1- organic acidemia and aminoacidopathy in 122 patients that 15.6% had normal development, 9.8% regression, 54.1% developmental delay, 20.5% developmental delay plus regression, 2-storage disease in 37 patients (10.8% of these patients had normal development, 32.4% regression, 32.4% developmental delay, 24.3% developmental delay plus regression 3- leukodystrophy in 27 patients (3.7% of these patients had normal development, 29.6% regression, 33.3% developmental delay, 33.3% developmental delay plus regression), other classes consisted of lipid oxidation disorders, urea cycle disorders, progressive myoclonic epilepsy; peroxizomal disorders in 27 patients (4 patients had normal development, 7 patients had regression, 14 of these patients had developmental delay and two people had developmental delay plus regression.

Totally, in developmental assessment 87% of patients showed neuro developmental delay/regression (consisted of 47.4% developmental delays; 21.1% developmental delays and regression and 18.3% developmental regression.) and only 13% of patients had normal development. Normal developmental status was seen in girls more than boys (Risk estimate: odds ratio for sex (female/male)=1.57). Normal developmental status was seen more on MCAD (8 patients) and homocystinuria (7 patients) disorders. Abnormal EEG was seen in 10.7% patients with normal development and 22.3% of patients with abnormal development (Odds ratio for EEG finding (NL/Abnormal) =2.38 with confidence interval 95%. MRI showed normal pattern in 10.7% of patients with normal development and 30% of patients with abnormal developmental status. Behavioral disorder was detected in 16.9% of patients. On examination, positive points in order of frequency consisted of: Abnormal muscular tonicity in 49.8% of patients, and ophthalmological problems in 32.9% of patients. Decreased level of consciousness episodes was seen in 5.2% etc. (Table1). 

**Table 1:** Positive Para Clinical Findings in Our Patients in This Study

	**n**	**%**
**High Lactate **	39	18.3
**High Ammonia **	29	13.6
**High Liver enzymes **	24	11.3
**Anemia **	23	10.8
**Acidosis **	22	10.3
**Abnormal HpLC **	15	7.0
**Low BS **	2	0.9
**High CSF/Pro **	1	0.5
**Low Calcium **	1	0.5

A total of 16% of patients were microcephaly and 12.7% were macrocephaly. Atotal of 55.5% of patients had seizures, of which the most common type was tonic and then GTC. Auditory problems were seen in 6.6% of patients. Positive points in paraclinical assessments in patients in order of frequency consisted of: increased ammonia and lactate increased liver enzymes, etc. (Table 1).

**Fig 2:**
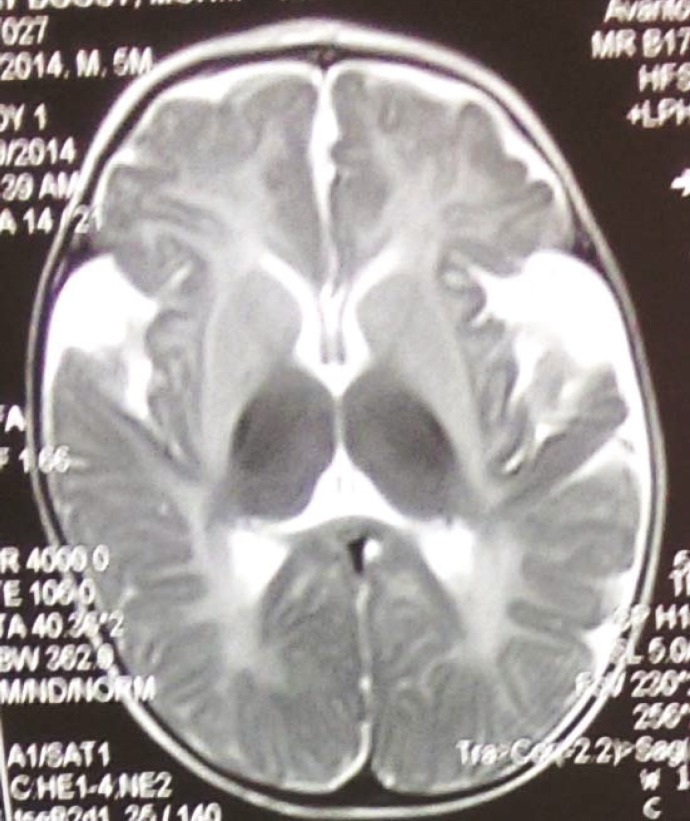
A 6-month-old boy-with Glutaric aciduria type1 with sylvian fissure widening in axial brain MRI

**Fig 3:**
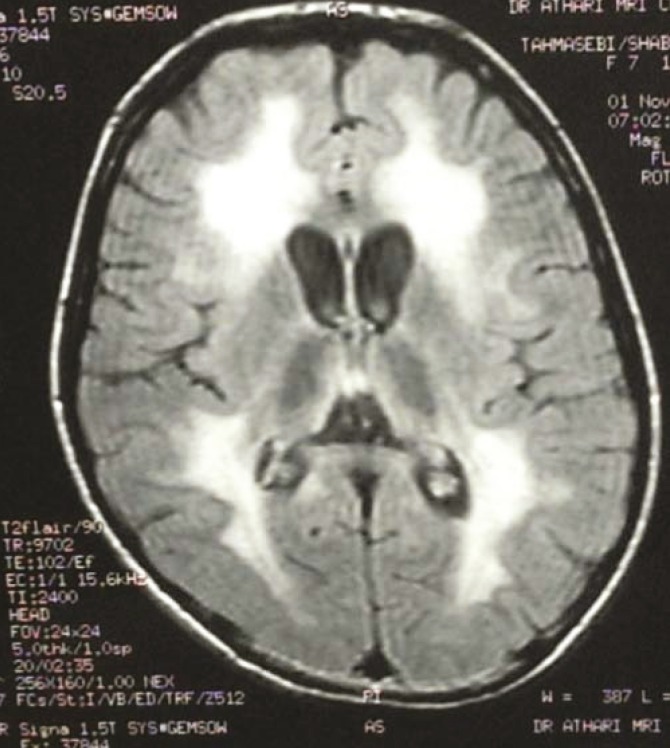
An 8-yr old-girl with metacromatic leuckodystrophia with periventricular white matter involvement (Butter-fly pattern) in imaging

Brain imaging in 36% of patients was normal; 20.4% of patients had brain atrophy; 25.1% had white matter involvement; 4.3% had basal ganglia involvement; 2.8% had basal ganglia and white matter involvement; 2.4% had basal ganglia involvement and brain atrophy. Brain atrophy with white matter involvement was seen in 5.7% of patients. In patients with organic acidemia and aminoacidopathy (including methyl malonicacidemia, Propionic acidemia, isovalericacidemia, glutaricaciduria I and II, MSUD, NKH, tyrosinemia, galactosemia, biotinidase deficiency, PKU, hemocysteinuria and MTHFR); in 33% of patients, brain imaging was normal; 28.9% of patients had brain atrophy; 24.5% of patients had white matter involvement; the rest of patients had basal ganglia along with other area involvement. For example, among 22 patients with glutaricaciduria, 20 patients had brain atrophy especially in fronto temporal regions with sylvian groove widening (Figure 2); 6 patients had white matter involvement and 6 patients had basal ganglia involvement. In 32 patients with PKU, 17 had white matter involvement and 6 patients had brain atrophy plus white matter involvement and Wilson’s disease); 55.6% had normal brain MRI; 11% had brain atrophy; 11% had white matter involvement; 8.3% had basal ganglia involvement and the remaining of patients had involvement in different regions. For example, 18 patients had GM2 gangliosidosis (Sandhoff and Tay–Sachs disease) that bilateral thalamus involvement in 5 patients; generalized brain atrophy in 5 patients; and white matter involvement as myelination delay in 3 patients were seen. In patients with urea cycle disorders, 33.3% had brain atrophy and 66.7% had white matter involvement. 22.2% of patients with peroxisomal disorders including ALD and Zellweger syndrome had normal brain MRI; 55.6% had white matter involvement; 11% had structural disorder and 11% had subdural hematoma. 72.7% of patients with fatty acid oxidation disorders including medium chain acyl coa deficiency (MCAD), long chain acyl coa deficiency (LCAD), short chain acyl coa deficiency (SCAD), and multiple carboxylase had normal brain MRI; 18.2% had brain atrophy, and 9% had basal ganglia involvement. In leukodystrophic disorders including MLD, Krabbe disease, Canavan, PMD, PMD-like; 7.4% of patients had normal imaging; 3.7% had brain atrophy; 81.5% had white matter involvement (Figure 3); 3.7% had basal gangliaand white matter involvement and 3.7% had white matter involvement and brain atrophy. 

For example, all the patients with Canavan disease (17 patients) had extensive white matter and u-fiber involvement, one patient had subdural effusion. From 4 patients with MLD, all of patients had white matter involvement as leukodystrophy with bilateral periventricular involvement without u-fiber involvement. Thirty six percent of patients with progressive myoclonic epilepsy (including NCL1, NCL2, Melas disease and Leigh disease), had normal imaging; 20.4% of patients had brain atrophy; 25.1% had white matter involvement, and the rest of patients had involvement in different regions. MRS was done in some of these patients, consisting of 17 patients with canavan disorder (that showed elevated level of N-acetyl aspartic acid), 3 patients with mitochondrial disorder (that showed elevated level of lactate), 4 patients with leukodystrophy in Brain MRI (that showed elevated level of choline/NAA ratio).

**Fig 4:**
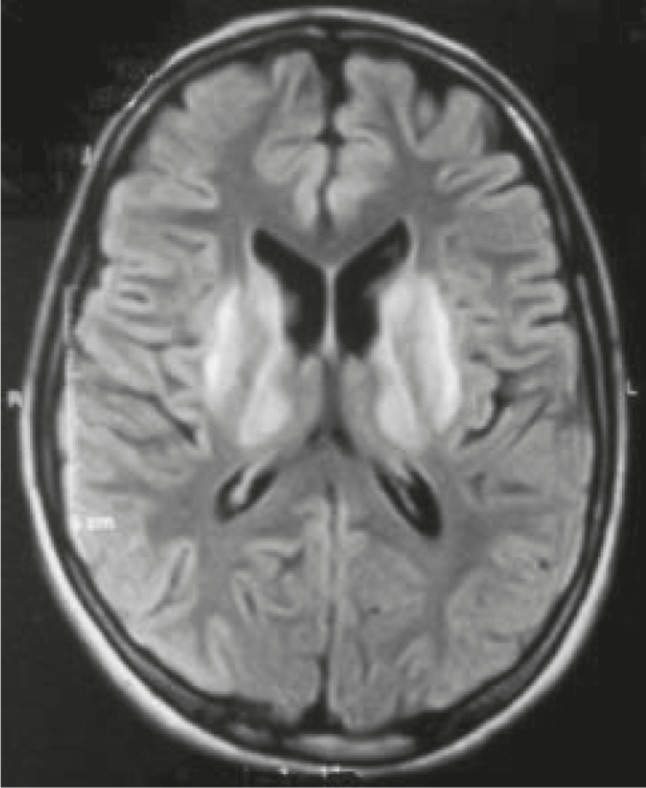
A 13-yr-old girl with wilson disease that showed basal ganglia involvement in brain imaging

Finally, patients who were fulfilled a number of our practical criteria items consisting (Table 2): Consanguinity of marriage, history of the same disorder or death in sibling, abnormal nurological exam, specific clinical findings, abnormal developmental status, seizure, abnormal EEG and abnormal brain imaging with specific patterns, were assessed as neurometabolicdisorders with different diagnostic method in referral lab (such as enzyme study, acylcarnitine profile, genetic study etc.). Out of 213 patients, 122 had organic acidemia and aminoacidopathy, 37 had storage disease, 27 had leukodystrophy, 27 had lipid oxidation disorders, urea cycle disorders, and progressive myoclonic epilepsy or peroxizomal disorders. Therefor the result of neuromrtabolic assessments as mention above, confirmed our practical criteria.

**Table 2:** Practical criteria for detection of neurometabolic patients

**History**	**Consanguinity of marriage (71.4%)**	**History of the same disorder or death in sibling,...(23.5%)**		
**Neurological exam**	Macrocephaly (18.8%)	Microcephaly (25.4%)	Hypotonocity or hypertonocity (49.8%)	Behavioral disorder (16.9%)
**Clinical**	Visual impairment (32.9%)	Hearing impairment (6.6%)	Dysmorfic feather (13.1%)	Abnormal skin and hair (20.7%)
**Developmental status**	Delay (47.4%)	Regression (39.4%)		
**seizure**	Current seizure (55%)	EEG abnormality (20.8)	Refractory seizures (38%)	
**Neuroimaging**	Abnormal MRI (64%)	Abnormal MRS (11.2%)		

## Discussion

71.4% of parents of our patients were relative (71.4%) and 23.5% of patients had positive family history of similar metabolic disordes. that in the other articles, the same results were noted. For example total of patients with MLC had history of consanguinity of marriage (7). Besides, 75% of 24 patients with hereditary metabolic disordes had history of consanguinity of marriage in their parents, and positive family history of similar metabolic disordes in 34% of first-degree relative (8). Seizures are one of the important presentations in neurometabolicdisordes. 36.5% % of our patients presented with seizure. Cause of 7% of neonatal seizures was metabolic disorders (9). Because metabolic disordes often involved the brain, there for, epilepsy same as other neurologic manifestations is common in these diseases (10). Assessment on 220 children with epilepsy in the first two years of life appeared and in 24 of these cases the etiology of seizures was metabolic disorders (8). Eight seven pecent of our patients illustrated developmental delay or developmental regression. Neurometabolic disorders can present with developmental delay and regression and the symptoms can appear at any age from newborns into adulthood (11). Developmental delay is a major symptom of various metabolic disorders that appear at the young age. So, understanding the general characteristics of developmental delay caused by metabolic disorders is an essential step for better identification and appropriate follow-up with metabolic workup (12). Bilateral thalamus involvement in patients (Turkish mustache pattern) and brain atrophy were seen in ourpatients with GM2 gangliosides (Sandhoff disease and Tay-Sachs disease). Brain MRI in patients with GM2 gangliosides show white matter abnormalities and changes in myelination of white matter that may be due to neuronal damage (13, 14). Our patients with glutaricaciduria had fronto temporal atrophy and sylvan fissure widening. Sylvan fissure widening and abnormalities in the basal ganglia, atrophy, and leukoencephalopathy has been reported in glutaricaciduria type 1 (15, 16). We found heterotopia and cortical malformation in our patients with Zellweger syndrome. Delayed myelination, leukoencephalopathy, and brain atrophy has been reported in Zellweger syndrome (17). Our patients with MLD had white matter involvement as periventricular leukodystrophy (a butter-fly pattern) without u-fiber involvement. Groeschel et al. reported early radiologic findings for this disorder including the corpus callosum and central white matter; cerebral, pons and cerebellum atrophy were involved as late sings. Furthermore, there was an association between cerebellar changes and u-fiber involvement (18). Brain edema and atrophy were seen in our patients with MSUD. Abnormal signal in the white matter and signal changes in the internal capsule of dorsal limb, cerebral hemisphere, and brain stem has been reported in MSUD patients (19, 20). Our phenylketonuria patients had white matter involvement. The white matter involvement due tointracellular hydrophilic metabolite accumulation has been revealed in patients with this disorder ( 21, 22). Leukodystrophic changes were found in our patients with Krabbe disease. Krabbe patients in the early stage of disease have increased dentate/cerebellar white matter intensity. Late onset patients, have deep cerebral white matter involvement in the posterior corpus callosum (23). Our patients with adrenoleukodystrophy (ALD) had leukodystrophic changes mostly in the posterior brain. In patients with ALD disorder, asymmetrical occipital white matter involvement is reported that is in the characteristic appearance of this disorder, as it progresses in a rostro-caudal direction (24). The basal ganglia involvement was seen in our patients with Wilson’s disease (Figure 4). Bilateral putaminal and thalamic involvement has been noted in Wilson’s disease. Of course, radiologic findings may be varied from focal thalamic lesions and dilatation of the third ventricle (25, 26). Our patients with NCL showed generalized brain and cerebellum atrophy in their brain MRI. Stroke and brain atrophy were seen in our patients with hemocysteinuria. Brain atrophy in patients with disorders of amino acid metabolism such as hemocysteinuria has frequently been reported (27, 28). Our patients with Canavan disease had wide white matter involvement with u-fiber involvement. In patients with Canavan disease white matter changes with a characteristic sub cortical U fibers involvement in thebrain MRI has showen (29, 30). However, Canavan disease without significant white matter involvement and diffuse abnormalities in cortex has been seen as well (31). An elevated level of N- acetyl aspartic acid was seen in patients with Canavan disease in MRS (magnetic resonance spectroscopy). Corpus callosum agenesis was found in patients with NKH. This is an autosomal recessively inherited disorder caused by an accumulation of glycine. Abnormalities in the corpus callosum development has noted in NKH (32). Our patients with propionic acidemia had brain atrophy and basal ganglia involvement. Bilateral basal ganglia involvement and severe neurological damage in patients with propionic acidemia has been reported (33, 34). Brain atrophy was seen in our patients with biotinidase deficiency. Ventriculomegaly and subdural effusion has been reported in patients with biotinidase deficiency (35, 36). Our patients with methylmalonicacidemia had brain atrophy and basal ganglia involvement. Mild to moderate myelination defect was seen in patients with methylmalonicacidemia, also basal ganglia involvement (in the globuspallidus) was found in these patients (37, 38). White matter involvement with hypomyelination was seen in our patients with PMD and PMD-like diseases. However, according to difficulties and less clinical clues in diagnosis of neurometabolic disorders, and that there is no criteria for diagnosis, our practical criteria, as the first criteria for detection of neurometabolic disorders, can be very useful and reduce the costs of diagnostic processes.

**In conclusions,** we suggest that patients, who have our criteria, are highly suspected to neurometabolic disorders, and should be assessed for these disorders.
